# Therapeutic and Diagnostic Roles of MSC‐Derived Exosomes in Alzheimer's Disease

**DOI:** 10.1002/brb3.71112

**Published:** 2025-12-07

**Authors:** Vinay Shankar Patil, Bhavin Parekh, Amit Sharma, Husni Farah, Renuka Jyothi‐S, Swati Mishra, Anima Nanda, Shaker Al‐Hasnaawei, Manoj Kumar‐Mishra

**Affiliations:** ^1^ Arnold and Marie Schwartz College of Pharmacy, Long Island University Brooklyn New York USA; ^2^ Faculty of Allied Medical Sciences, Hourani Center For Applied Scientific Research Al‐Ahliyya Amman University Amman Jordan; ^3^ Department of Biotechnology and Genetics, School of Sciences JAIN (Deemed to Be University) Bangalore Karnataka India; ^4^ Department of Pharmacology IMS and SUM Hospital, Siksha ‘O’ Anusandhan Bhubaneswar Odisha India; ^5^ Department of Biomedical Sathyabama Institute of Science and Technology Chennai Tamil Nadu India; ^6^ College of Pharmacy the Islamic University Najaf Iraq; ^7^ Department of Medical analysis, Medical laboratory Technique College the Islamic University of Al Diwaniyah Al Diwaniyah Iraq; ^8^ College of Business and Economics Salale University Fitche Ethiopia

**Keywords:** Alzheimer's disease, biomarkers, exosomes, mesenchymal stem cells, neuroregeneration

## Abstract

**Purpose:**

Alzheimer's disease (AD), the leading cause of dementia, is characterized by amyloid‐β accumulation, tau hyperphosphorylation, neuroinflammation, and synaptic failure, with no curative therapies available. This review aims to explore innovative therapeutic and diagnostic strategies, focusing on mesenchymal stem cell–derived exosomes (MSC‐exos) as potential disease‐modifying agents.

**Method:**

The review synthesizes current evidence on the regenerative, immunomodulatory, and neuroprotective properties of mesenchymal stem cells (MSCs) and their exosomes. It examines how MSC‐exos, as nanosized extracellular vesicles carrying proteins, lipids, and nucleic acids, interact with the central nervous system to modulate disease pathways.

**Findings:**

MSC‐exos can cross the blood–brain barrier (BBB), deliver neurotrophic factors, modulate microglial activity, enhance amyloid clearance, and support neuronal survival and synaptic plasticity. They also hold promise as biomarkers by reflecting central nervous system pathology in peripheral biofluids. Early clinical trials using MSCs from bone marrow, adipose tissue, and umbilical cord show safety and feasibility, with exosome‐based approaches offering scalable, cell‐free alternatives.

**Conclusion:**

MSC‐derived exosomes present a promising avenue for both therapeutic intervention and early diagnosis in AD, offering neuroprotective, anti‐inflammatory, and pro‐regenerative effects. However, further progress requires addressing challenges such as exosome isolation standardization, cargo characterization, and regulatory considerations to enable their translation into clinical practice.

## Graphical abstract

Mesenchymal stem cell (MSC)‐derived exosomes deliver bioactive cargos, including miRNAs, neurotrophic factors, and antioxidant molecules, that modulate neuronal survival, microglial phenotype, and amyloid plaque clearance in Alzheimer's disease. These actions reduce apoptosis, oxidative stress, and inflammation, promote neurogenesis, enhance Aβ degradation, and ultimately lead to therapeutic outcomes such as reduced amyloid burden, improved synaptic plasticity, enhanced cognition, and long‐term neuroprotection.

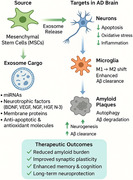

**Abbreviations**
5XFAD: five‐mutation familial AD mouse model
AD: Alzheimer's disease
ADE: astrocyte‐derived exosome
Akt: protein kinase B
APP: amyloid precursor protein
APP/PS1: mouse model expressing mutant APP and PSEN1
Aβ: amyloid‐β
BACE‐1: β‐site APP cleaving enzyme‐1
BBB: blood–brain barrier
BDNF: brain‐derived neurotrophic factor
CAA: cerebral amyloid angiopathy
CSF: cerebrospinal fluid
GFAP: glial fibrillary acidic protein
HGF: hepatocyte growth factor
IDE: insulin‐degrading enzyme
LTP: long‐term potentiation
MSC‐exo: mesenchymal stem cell–derived exosome
MSC: mesenchymal stem cell
NDE: neuron‐derived exosome
NEP: neprilysin
NF‐κB: nuclear factor kappa‐B
NGF: nerve growth factor
PET: positron emission tomography
PI3K: phosphoinositide 3‐kinase
PSD‐95: postsynaptic density protein‐95
RVG: rabies virus glycoprotein
VEGF: vascular endothelial growth factor

## 1 Introduction

Alzheimer's disease (AD) is the most prevalent form of dementia and a progressive neurodegenerative disorder with major global impact (A. Kumar, Sidhu, et al. 2024). Age is the strongest predictor of onset; about one in nine individuals over 65 is affected, and more than six million people in the United States live with AD (2023 Alzheimer's Disease Facts and Figures 2023). Since Alois Alzheimer's first description in 1906, diagnostic criteria have evolved to combine clinical assessment with biomarker‐based approaches that enable identification of preclinical stages (2023 Alzheimer's Disease Facts and Figures 2023; Qiu et al. 2009). With rising life expectancy, projections estimate up to 152 million cases by 2050, underscoring a mounting public health challenge (Qiu et al. 2009). In the United States, AD is the fifth leading cause of death, and mortality attributed to AD has more than doubled since 2000, highlighting the need for earlier detection and more effective interventions beyond symptomatic relief (2023 Alzheimer's Disease Facts and Figures 2023; Chaddha et al. 2025).

Neuropathologically, AD features extracellular amyloid‐β (Aβ) deposition and intracellular hyperphosphorylated tau, which contribute to synaptic dysfunction and neuronal loss in memory‐critical regions such as the hippocampus and cortex (DeTure and Dickson 2019; Mehrnoosh et al. 2025). Clinically, patients develop progressive deficits in memory, executive function, and language that culminate in loss of independence (Safiri et al. 2024). Non‐age risk factors include cardiometabolic disease, Down syndrome, and lifestyle elements such as inactivity and poor diet (Edwards et al. 2019). Mechanistically, altered processing of amyloid precursor protein (APP) with accumulation of Aβ and tau hyperphosphorylation triggers neuroinflammation, vascular dysfunction, and synaptic failure that drive degeneration (Kamatham et al. 2024; Tenchov et al. 2024).

Current drugs such as donepezil and rivastigmine offer modest symptomatic benefit and do not halt progression, while side effects can limit use in older adults (Passeri et al. 2022; Abbas Raja et al. 2025). Consequently, multi‐target strategies are under investigation, including immunomodulation, anti‐amyloid and anti‐tau approaches, and regenerative modalities. Mesenchymal stem cell–derived exosomes (MSC‐exos) are particularly promising because they carry bioactive cargos with anti‐inflammatory and neurotrophic actions, show low immunogenicity, and can be engineered for delivery to the central nervous system (Z. J. Ma et al. 2020; Shah et al. 2024; Pinky et al. 2021). This review outlines AD pathophysiology to frame the therapeutic and diagnostic potential of MSC‐exos and summarizes ethical and regulatory considerations relevant to translation.

## 2 Pathophysiology of AD

Aβ peptides, particularly Aβ42, are generated by β‐ and γ‐secretase cleavage of APP. They aggregate into oligomers and plaques that disrupt synaptic transmission and activate microglia and astrocytes, which initiate inflammatory cascades (Grewal et al. 2025). In parallel, tau normally stabilizes microtubules but in AD becomes hyperphosphorylated, detaches, misfolds, and forms neurofibrillary tangles that impair axonal transport and promote neuronal death (W. Zhang et al. 2023). These molecular abnormalities are accompanied by cholinergic deficits, glial activation, and progressive atrophy in hippocampal and cortical networks that are essential for learning and memory (Sheppard and Coleman 2020).

**FIGURE 1:**
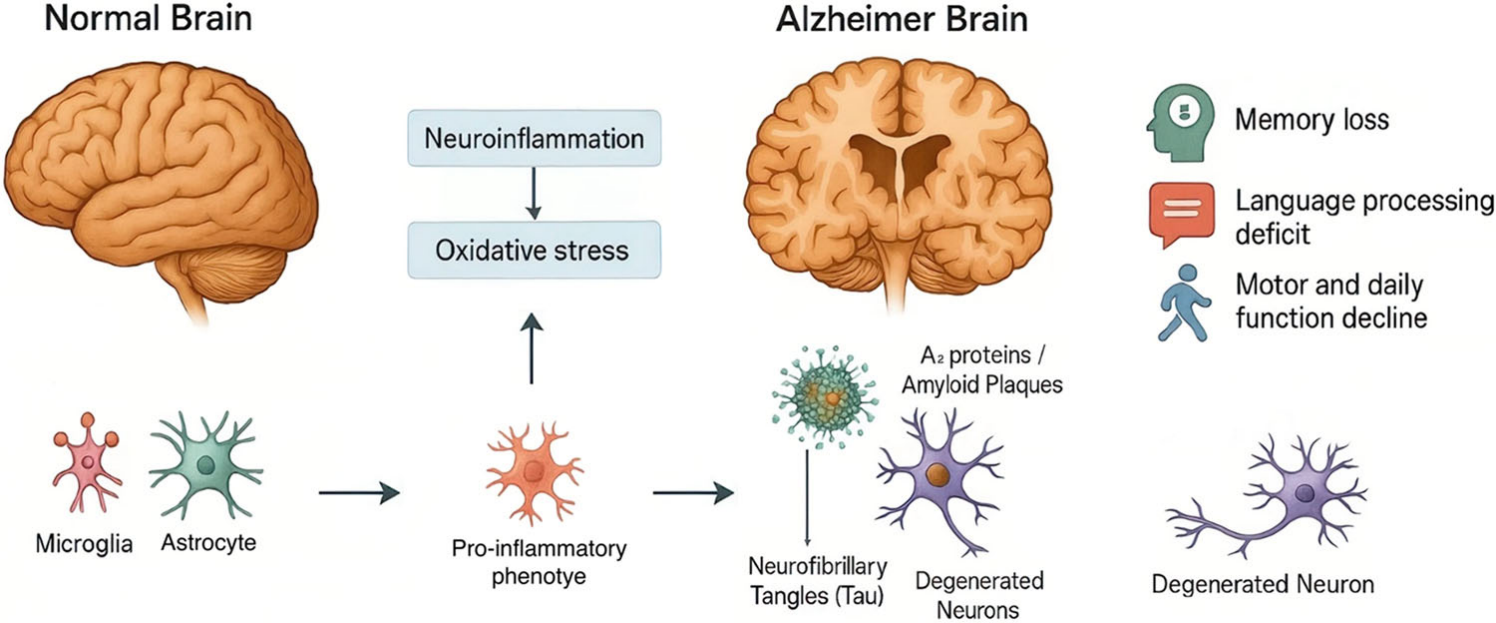
AD is marked by Aβ plaques, tau tangles, and severe neuronal degeneration. These pathological changes lead to cortical atrophy, memory loss, and impaired cognitive function.

Several partially overlapping hypotheses describe disease initiation and spread. The cholinergic hypothesis attributes cognitive decline to loss of cholinergic neurons and acetylcholine, which can amplify the impact of Aβ on synaptic communication (Z. R. Chen et al. 2022). The amyloid hypothesis proposes that accumulation of Aβ, especially Aβ42, triggers downstream tau pathology, synaptic failure, and neurodegeneration (X. Zhang et al. 2018). Braak et al. (2011) described a six‐stage framework that maps a stereotyped progression of tau pathology and correlates more closely with dementia severity than amyloid plaque burden, which supports the idea that amyloid may initiate pathology while tau better tracks neurodegeneration (Bloom 2014). Cerebral amyloid angiopathy (CAA) further contributes to vascular dysfunction and cognitive decline (Greenberg et al. 2020) (Figure 1).

In sum, converging processes that include Aβ accumulation, tau dysregulation, synaptic injury, neuroinflammation, and vascular pathology create a multifactorial landscape that supports the rationale for multi‐node interventions such as mesenchymal stem cell (MSC) exosome strategies (Kamatham et al. 2024; Bhatti et al. 2023). Table 1 links these hallmarks to exosome relevance (Checler et al. 2021; Rawat et al. 2022; Cai et al. 2022; de Godoy et al. 2018; Govindpani et al. 2019).

**TABLE 1:** Pathophysiological hallmarks of AD and exosome relevance.

Hallmark of AD	Molecular mechanism	Exosome‐associated role	Consequence in CNS	Therapeutic implication	Ref
**Aβ aggregation**	APP cleavage by β‐ and γ‐secretases → Aβ42 oligomers	Exosomes carry APP, BACE‐1, Aβ peptides	Plaque formation, synaptic dysfunction	MSC‐exos may enhance Aβ clearance via microglia	Checler et al. (2021)
**Tau hyperphosphorylation**	Abnormal phosphorylation of tau → neurofibrillary tangles	Exosomes transport tau seeds between neurons	Cytoskeletal collapse, neuronal death	Potential target for exosome‐mediated tau degradation	Rawat et al. (2022)
**Neuroinflammation**	Microglial/astrocytic activation → cytokine storm	Exosomes modulate M1/M2 microglial polarization	Exacerbated neuronal damage	MSC‐exos deliver anti‐inflammatory miRNAs (IL‐10, IL‐4)	Cai et al. (2022)
**Synaptic dysfunction**	Loss of cholinergic signaling, oxidative stress	Exosomes regulate synaptic proteins (PSD‐95, synaptophysin)	Impaired learning and memory	Exosome therapy restores synaptic plasticity	de Godoy et al. (2018)
**Vascular dysfunction**	CAA, hypoperfusion	Exosomes influence angiogenesis (VEGF, HGF)	Reduced nutrient/oxygen delivery	MSC‐exos stimulate angiogenesis and perfusion	Govindpani et al. (2019)

## 3 Neuroregeneration Therapy

### 3.1 Stem Cells

Stem cells are defined by self‐renewal and multipotency and have been investigated for neurological disorders, with encouraging signals first seen in transplantation studies for Parkinson's disease (Shao et al. 2025; Hussen et al. 2024). Beyond cell replacement, transplanted stem cells release trophic and immunomodulatory factors that support neuronal survival and recruit endogenous precursor cells, thereby promoting repair (Y.‐T. Wang and Yuan 2022). While pluripotent platforms such as hESCs and induced pluripotent stem cells (iPSCs) offer theoretical scalability, their clinical translation in neurodegeneration is constrained by immune, ethical, and tumorigenicity concerns and by manufacturing complexity; hence, their discussion here is kept brief (Volarevic et al. 2018; Matoba and Zhang 2018; Romito and Cobellis 2016; Wuputra et al. 2020).

In AD, MSCs and their secreted exosomes have emerged as the most practical regenerative avenue because MSCs are accessible from bone marrow, adipose tissue, and umbilical cord, exhibit low immunogenicity, and possess robust paracrine activity (Deokate et al. 2024; Kulus et al. 2021; X. Han et al. 2025). MSC‐exos carry proteins, lipids, and nucleic acids that can modulate disease pathways. In AD models, MSC‐exos mitigate neuroinflammation, support synaptic function, and facilitate amyloid clearance, consistent with their cargo of neurotrophic and anti‐inflammatory mediators (Shah et al. 2024). Targeted and noninvasive delivery strategies strengthen this rationale. Conjugation of MSC‐exos with the rabies virus glycoprotein (RVG, neuronal‐tropic peptide) peptide increases neuronal tropism in APP/PS1 (mouse model expressing mutant APP and PSEN1) mice, with greater hippocampal and cortical accumulation, reduced astrocyte activation and amyloid burden, and improved performance in the Morris water maze, together with a shift toward anti‐inflammatory cytokine profiles (Sun et al. 2021; Cui et al. 2019). Intranasal administration of small extracellular vesicles produced by three‐dimensional cultures of bone marrow MSCs improves learning and memory in 5XFAD (five‐mutation familial AD mouse model) mice, lowers plaque burden in the hippocampus, and reduces colocalization of glial fibrillary acidic protein (GFAP) with amyloid, suggesting concurrent anti‐amyloid and anti‐inflammatory effects (Cone et al. 2021).

Mechanistically, MSCs and their exosomes promote anti‐inflammatory signaling, stimulate neurogenesis through pathways such as Wnt, and enhance proteostasis. In vitro and in vivo studies indicate reductions in toxic Aβ42 that align with increased autophagy and lysosomal activity, while synaptic markers and neuronal survival improve in parallel (Planat‐Benard et al. 2021; Oh et al. 2015; Qin et al. 2022). Collectively, these data support MSC‐exos as a cell‐free, immunologically compatible modality that can engage multiple AD nodes from inflammation to synaptic resilience, and they provide a focused foundation for the clinical perspectives detailed below.

### 3.2 Exosomes

#### 3.2.1 Isolation of Exosomes

Several methods are currently employed for the isolation of exosomes, each with distinct advantages and limitations:

- *Ultracentrifugation*: This remains the most commonly used and conventional technique for exosome isolation from stem cell cultures and biofluids. Initial low‐speed spins remove cells and debris, while subsequent high‐speed ultracentrifugation pellets crude exosomal fractions. These preparations may be used directly or further refined by density gradient ultracentrifugation to improve purity (Coughlan et al. 2020).
- *Size‐based filtration*: Exosomes can also be enriched by passing samples through filters with defined pore sizes or by size‐exclusion chromatography, thereby eliminating larger vesicles (> 150 nm) and smaller particles (< 50 nm). Although this method ensures size uniformity, it is not sufficient for true enrichment; ultracentrifugation is often applied as a complementary step (Doyle and Wang 2019).
- *Polymer precipitation*: Precipitation with hydrophilic polymers such as polyethylene glycol (PEG) reduces exosome solubility, forcing them to sediment within the exosomal size range (30–150 nm). This method is simple and feasible with standard laboratory equipment; however, yield and purity strongly depend on polymer size and concentration (Chavda et al. 2023; Al‐Sahlawi et al. 2024).
- *Immunoaffinity capture*: Exploiting the presence of specific surface proteins on exosomes, this method employs antibodies coupled to agarose beads or magnetic particles to selectively isolate subpopulations with high purity. Immunoaffinity is widely used in both research and clinical applications, including biomarker discovery and diagnostic assays. Its limitations include high cost and dependence on available antibody reagents (De Sousa et al. 2023).

#### 3.2.2 Cell Culture

MSCs, one of the main sources of therapeutic exosomes, require carefully controlled culture conditions to maintain viability and functionality. Typically, MSCs are grown in Dulbecco's Modified Eagle Medium (DMEM) supplemented with fetal bovine serum (FBS), which provides essential nutrients and growth factors. To prevent microbial contamination, antibiotics and antifungal agents are added. Cultures are maintained at 37°C in a humidified atmosphere containing 5% CO_2_, conditions that closely mimic the physiological environment (Z. J. Ma et al. 2020; H. Yu, Zhang, et al. 2025). For translational applications, however, it is critical to minimize or completely eliminate animal‐derived components. Thus, serum‐free or chemically defined media are increasingly employed. Once MSCs reach the appropriate confluence, they can be expanded or harvested for exosome production, ensuring that they remain healthy and retain their multipotent differentiation potential (Wa et al. 2024).

#### 3.2.3 Biogenesis, Secretion, and Uptake

Exosome formation follows a highly orchestrated sequence of events beginning with endocytosis and culminating in vesicle release. Initially, early sorting endosomes (ESEs) are generated through plasma membrane invagination, incorporating extracellular molecules and surface proteins (Q. F. Han et al. 2022). During endosome maturation, the inward budding of the limiting endosomal membrane produces intraluminal vesicles (ILVs), which accumulate within multivesicular bodies (MVBs) (Y. Wang, Xiao, et al. 2024). The endosomal sorting complex required for transport (ESCRT) machinery is the primary regulator of this process. Comprising ∼30 proteins, the ESCRT system is organized into four complexes (ESCRT‐0, ‐I, ‐II, ‐III) and associated proteins (Vps4, Alix, Tsg101). Together, they coordinate ubiquitinated cargo recognition, membrane deformation, vesicle scission, and recycling of ESCRT components (C. Wang, Chen, et al. 2023).

**FIGURE 2:**
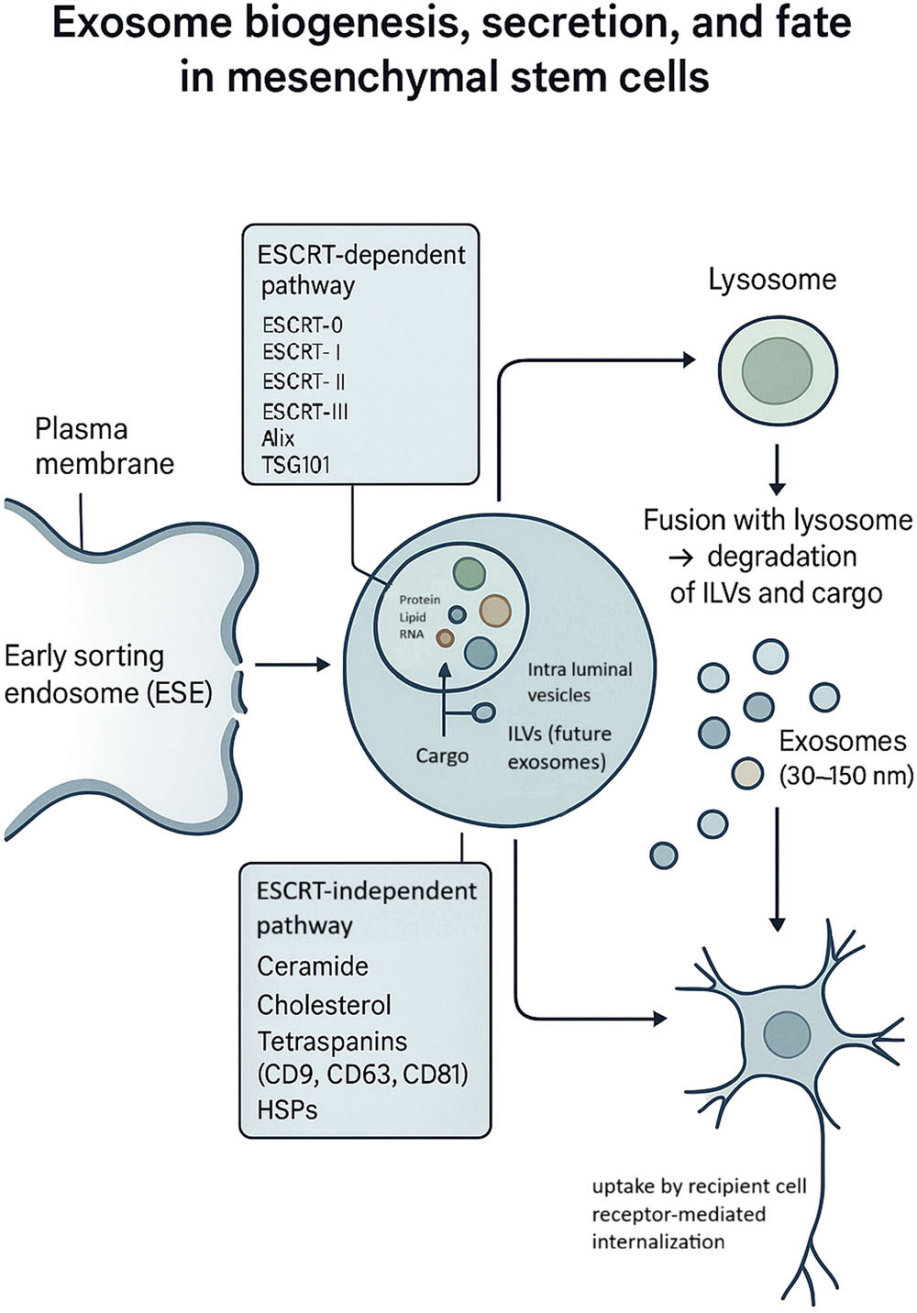
Exosome biogenesis, secretion, and fate in mesenchymal stem cells. Early sorting endosomes mature into multivesicular bodies containing intraluminal vesicles (future exosomes) that are loaded with proteins, lipids, and RNAs through ESCRT‐dependent and ESCRT‐independent pathways. These multivesicular bodies either fuse with lysosomes for degradation or with the plasma membrane to release 30–150 nm exosomes, which are then taken up by recipient cells via receptor‐mediated internalization.

In parallel, ESCRT‐independent pathways also contribute to exosome biogenesis. These involve tetraspanins, ceramides, cholesterol, phosphatidic acids, and heat‐shock proteins (HSPs), all of which regulate lipid reorganization and RNA cargo loading (C. Wang, Chen, et al. 2023). Cytoplasmic materials including RNA, proteins, and lipids are then packaged into ILVs, while the Golgi apparatus and endoplasmic reticulum provide additional input (Gurung et al. 2021). The fate of MVBs is bifurcated: some fuse with lysosomes or autophagosomes for degradation, whereas others are trafficked along the cytoskeletal network to the plasma membrane, where vesicle fusion releases exosomes into the extracellular milieu (Yadav et al. 2024). Ceramides are particularly enriched in secretory MVBs compared with degradative ones, suggesting that lipid composition influences vesicle destiny (Horbay et al. 2022). Exosomes are characterized by a distinct set of molecular markers. Protein markers include flotillin, Alix, TSG101, and tetraspanins (CD9, CD63, CD81), while lipids such as ceramide and sphingomyelin are highly concentrated, reflecting their lipid raft origin (Gurung et al. 2021) (Figure 2).

## 4 Exosomes as Biomarkers of AD

Current AD diagnostics rely on cerebrospinal fluid (CSF) biomarkers such as Aβ42/40, total tau, and phosphorylated tau, together with cognitive assessments and neuroimaging including positron emission tomography (PET) and CT. These standards have clear value but face limitations for population‐level screening and longitudinal monitoring, since CSF collection is invasive and imaging can be costly and variably interpreted. Because AD pathology often precedes symptoms by years, there is an unmet need for minimally invasive biomarkers that detect earlier disease stages (Hampel et al. 2023; Modat et al. 2023; X. Zhang et al. 2017; Staffaroni et al. 2017).

Exosomes are nanosized extracellular vesicles that encapsulate proteins, lipids, and nucleic acids reflective of their parent cells. In the nervous system, neuron‐derived exosomes (NDEs) display surface molecules that facilitate selective uptake by recipient cells. Crucially, exosomes can be isolated from accessible biofluids such as blood, urine, and saliva, and their stability after collection supports clinical workflows (M. A. Kumar, Baba, et al. 2024; Huo et al. 2021; Delshad et al. 2025). Reports on exosome concentration and size in AD are not fully consistent, which likely reflects differences in biofluid source, isolation method, and analytic pipelines. Convergence across studies will require standardized collection, isolation, and characterization protocols to clarify the diagnostic meaning of these morphological metrics (Soliman et al. 2021; Yakubovich et al. 2022).

**FIGURE 3:**
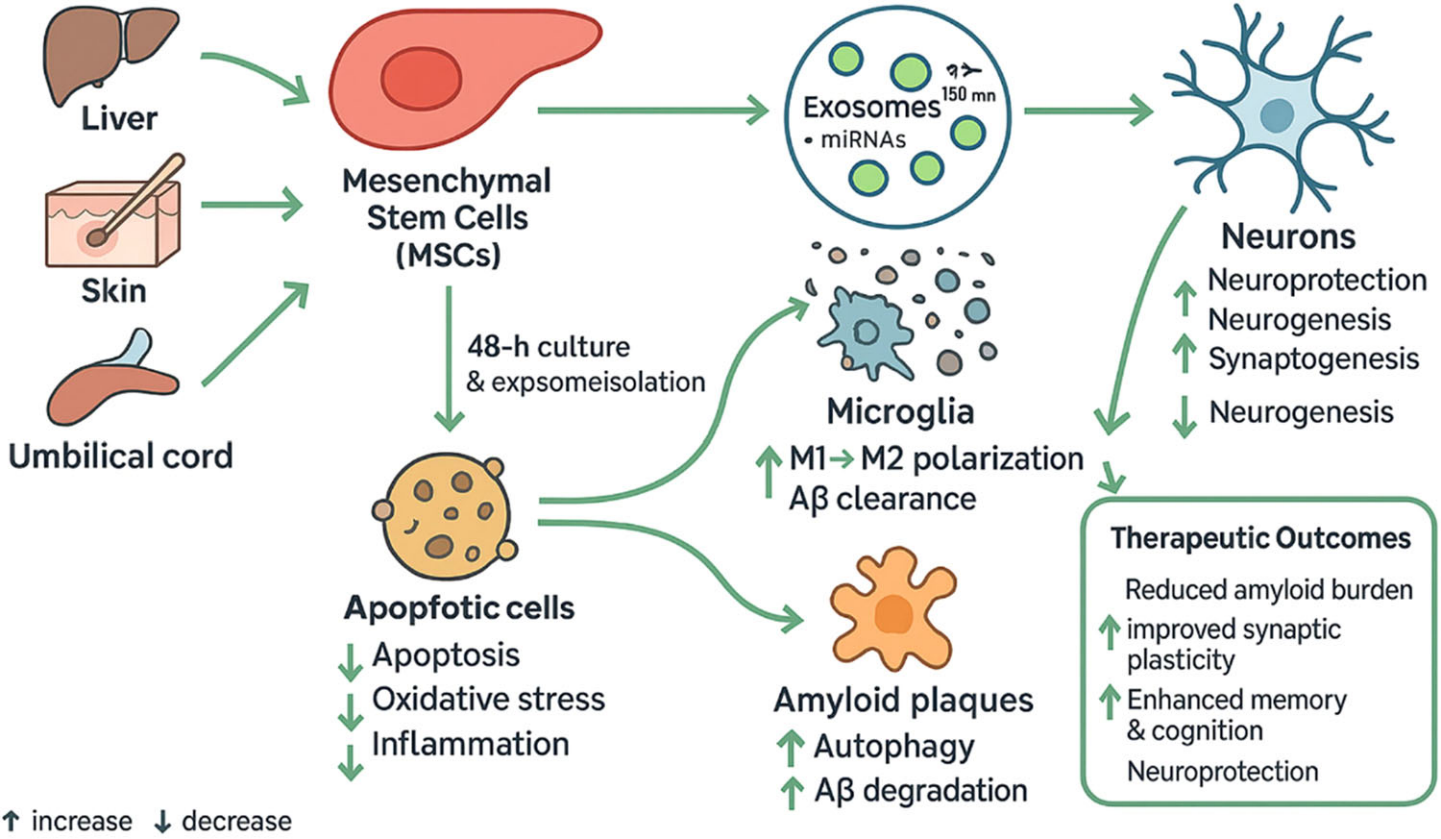
MSC‐derived exosomes deliver miRNAs, proteins, and neurotrophic factors to neurons and microglia in the AD brain, promoting neurogenesis, Aβ clearance, immune regulation, and neuroprotection. These effects collectively enhance synaptic plasticity and cognitive function.

Molecular cargo offers stronger translational traction. Proteomic analyses have identified AD‐linked proteins within exosomes, including β‐site APP cleaving enzyme‐1 (BACE‐1), sAPPα, sAPPβ, γ‐secretase components, and Aβ peptides, directly connecting vesicle content to amyloidogenic pathways (Zhao et al. 2023). Lipidomic data suggest that specific lipids and plasmalogen glycerophosphoethanolamines are enriched in brain‐derived exosomes from AD, providing a complementary signature (Ghadami and Dellinger 2023). Exosomal microRNAs also show reproducible alterations: panels measured in serum and CSF frequently detect upregulation of species such as miR‐15a‐5p and downregulation of others including miR‐15b‐3p and miR‐29c, consistent with disease‐related gene regulatory shifts (Bhome et al. 2018; Xia et al. 2019; Wei et al. 2020). By integrating exosomal markers with established amyloid and tau measures, composite signatures may improve diagnostic performance and staging while enabling less invasive, repeatable sampling (M. A. Kumar, Baba, et al. 2024; Dehghani et al. 2025). Figure 3 summarizes how MSC‐derived exosomes could deliver miRNAs, proteins, and neurotrophic factors to modulate neurogenesis, Aβ clearance, immune tone, and synaptic plasticity in the AD brain (Mukerjee et al. 2025).

Among blood‐based approaches, enrichment of brain‐derived exosomes has been proposed as a liquid biopsy for AD. Although their abundance in plasma is lower than in CSF, brain‐derived vesicles can cross the blood–brain barrier (BBB) and retain molecular information from their neuronal origins, enabling peripheral capture of central signals (Shah et al. 2024; A. Kumar, Nader, et al. 2024). Immunoprecipitation from plasma allows selective recovery of neuronal or glial exosomes and may reduce diagnostic overlap seen with conventional CSF thresholds of Aβ42/40, T‐tau, and p‐Tau181. Multicenter studies reporting correlations between protein levels in blood‐derived exosomes and CSF strengthen their potential as surrogate indicators of central pathology (Mukerjee et al. 2025; Guha et al. 2019).

NDEs carry synaptic and lysosomal proteins that have been associated with progression from mild cognitive impairment to dementia and with disease severity, supporting roles in early detection and longitudinal monitoring (Huo et al. 2021; Winston et al. 2016). Astrocyte‐derived exosomes (ADEs) contain complement proteins that vary by stage and often harbor higher levels of proteins relevant to amyloid processing than NDEs, highlighting astroglial contributions and potential therapeutic targets (Goetzl et al. 2018; Y. Yu, Wang, et al. 2025).

Translation to practice will require large, well‐controlled cohorts with harmonized pre‐analytics and analytics. Standardized operating procedures for isolation, quantification, and molecular profiling are essential to reproducibility and clinical adoption. Even so, current evidence positions exosome‐based assays as promising complements to CSF and imaging, with potential to enable earlier detection and more precise tracking of AD biology (Youssef et al. 2025; X. Li et al. 2019).

## 5 Exosome‐Based Therapeutics in AD

Beyond diagnostics, exosomes also hold significant therapeutic promise, particularly through their association with MSCs. Compared with iPSCs or neural stem cells (NSCs), MSCs are favored for their safety profile, versatility, and robust immunomodulatory capacity (M. A. Kumar, Baba, et al. 2024). They exert neuroprotective effects by releasing neurotrophic factors such as brain‐derived neurotrophic factor (BDNF) and vascular endothelial growth factor (VEGF), which promote neuronal survival, neurogenesis, and synaptic remodeling (Yari et al. 2022). MSCs also demonstrate low tumorigenic risk, are relatively easy to obtain from sources such as bone marrow and adipose tissue, and exhibit low immunogenicity, facilitating allogeneic transplantation without the need for extensive immunosuppression (Fan et al. 2020).

Through paracrine signaling and exosome release, MSCs deliver a diverse array of therapeutic molecules including growth factors, microRNAs, and membrane‐bound proteins. These mediators collectively inhibit apoptosis, reduce oxidative stress, and enhance neuronal regeneration (Nasirishargh et al. 2021). Neurotrophins secreted by MSCs, including VEGF, hepatocyte growth factor (HGF), nerve growth factor (NGF), and neurotrophin‐3, support neuritic outgrowth and functional recovery in damaged brain regions (M. Li et al. 2022). Importantly, MSC‐exos also regulate immune responses by shifting microglia away from a pro‐inflammatory M1 phenotype toward a neuroprotective M2 state. This dual action not only dampens chronic neuroinflammation but also promotes microglial clustering around amyloid deposits, thereby facilitating Aβ clearance through autophagic and lysosomal mechanisms (Miron et al. 2024).

Experimental studies further illustrate the versatility of MSC‐derived exosomes. For instance, exosomes secreted by hypoxia‐preconditioned MSCs have been shown to enhance learning and memory in transgenic AD mice, reduce amyloid accumulation, and elevate synaptic protein expression. These findings suggest that the therapeutic efficacy of MSC‐exos can be modulated by preconditioning strategies, reflecting their adaptability to different physiological environments (Tan et al. 2024). Similarly, soluble factors secreted by MSCs, such as galectin‐3 or ICAM‐1, have been shown to stimulate neprilysin (NEP) production in microglia, enhancing amyloid degradation. Collectively, these actions highlight the ability of MSC‐derived exosomes to alter the neurodegenerative microenvironment and support neuronal resilience (M. A. Kumar, Baba, et al. 2024; Shao et al. 2025).

Moreover, bone marrow–derived MSCs have been shown to recruit microglia through chemokine signaling (e.g., CCL5), inducing protective cytokines such as IL‐4 and neprilysin, which together reduce amyloid burden and improve cognitive outcomes. These mechanistic insights indicate that exosome‐mediated pathways are not passive byproducts of stem cell therapy but active contributors to its therapeutic benefits (Regmi et al. 2022). Importantly, ongoing research suggests that tailoring the properties of MSC‐derived exosomes, through genetic modification, preconditioning, or optimized culture conditions, may further enhance their efficacy in combating AD pathology (Liao et al. 2024).

MSC‐derived exosomes transport miRNAs, proteins, and enzymes that converge on canonical AD pathways (Oveili et al. 2023). For amyloid biology, exosomal miR‐29 family and miR‐107 have been linked to the regulation of APP and BACE‐1, while delivery of neprilysin and insulin‐degrading enzyme (IDE) supports proteolysis of Aβ. Autophagy and lysosome function are promoted through TFEB activation and cathepsin cargo, which facilitates clearance of misfolded proteins and amyloid species (Bécot et al. 2020). For tau pathology, miR‐132 or miR‐212 and miR‐146a have been associated with modulation of tau kinases and phosphatases, which can reduce tau phosphorylation and spread (Boscher et al. 2020).

Synaptic resilience is supported by exosomal neurotrophins such as BDNF and NGF that engage TrkB or ERK or phosphoinositide 3‐kinase (PI3K) or protein kinase B (Akt) signaling to enhance dendritic spine stability, long‐term potentiation (LTP), and expression of synaptic proteins including postsynaptic density protein‐95 (PSD‐95) and synaptophysin (Numakawa and Kajihara 2023). Neuroinflammation is attenuated through delivery of miR‐21, miR‐124, and miR‐146a, which downregulate nuclear factor kappa‐B (NF‐κB) activity and shift microglia from an M1 to an M2 phenotype with increased IL‐4 and IL‐10 and reduced TNF‐α and IL‐1β (Slota and Booth 2019). Anti‐apoptotic and antioxidant effects follow from activation of PI3K or Akt and upregulation of SOD and peroxiredoxins, which lowers oxidative stress (Y. Chen et al. 2021). Together, these mechanisms provide a biologically coherent basis for the behavioral and histologic improvements observed in preclinical models and suggest measurable endpoints such as neuron‐derived exosomal BACE‐1 and synaptic proteins, phospho‐tau to total tau ratios, and autophagy markers for early phase trials (Shiau et al. 2022). To integrate therapeutic and diagnostic perspectives, we map how MSC‐exosomal cargos engage specific pathways to modify *AD* processes and yield measurable readouts. This framework connects miRNA and protein cargos to Aβ clearance, tau regulation, synaptic repair, neuroinflammation, and neurovascular support, providing testable endpoints for trials, as summarized in Table 2.

**TABLE 2:** Cargo–pathway–disease process–readout framework for MSC‐derived exosomes in AD.

Exosomal cargo	Primary target or pathway	Disease process affected	Expected effect	Potential readout	Ref
**miR‐29, miR‐107**	APP or BACE‐1 regulation	Amyloidogenesis	Lower Aβ production	Plasma or CSF Aβ42/40 ratio; NDE BACE‐1	Lei et al. (2015), W. X. Wang et al. (2008)
**NEP, IDE, cathepsins**	Proteolysis or lysosome	Aβ clearance	Higher degradation and clearance	IDE activity; lysosomal markers in NDEs; amyloid PET	Grimm et al. (2013), Huang et al. (2025)
**TFEB axis components**	Autophagy or lysosome biogenesis	Proteostasis	Enhanced removal of misfolded proteins	LC3 or p62 ratios; NDE TFEB targets	X. Wang, Xie, et al. (2023), Xiao et al. (2015)
**miR‐132 or miR‐212**	Tau kinases or phosphatases	Tau phosphorylation	Lower p‐Tau and reduced spread	Plasma or CSF p‐Tau; tau PET	M. Zhang and Bian (2021)
**BDNF, NGF**	TrkB or ERK or PI3K or Akt	Synaptic plasticity	Higher LTP and synaptic proteins	NDE PSD‐95 or synaptophysin; cognitive composites	Liu et al. (2022)
**miR‐124, miR‐146a, miR‐21**	NF‐κB and microglial polarization	Neuroinflammation	M1 to M2 shift; lower TNF‐α and IL‐1β	ADE cytokine cargo; TSPO‐PET	Mavroudis et al. (2023), Wan et al. (2022)
**VEGF, HGF**	Angiogenesis or perfusion	Neurovascular unit	Improved cerebral perfusion	MRI perfusion measures	Fayazi et al. (2021), Manuel et al. (2017), Yari et al. (2022)

Abbreviations: AD, Alzheimer's disease; ADE, astrocyte‐derived exosomes; IDE, insulin‐degrading enzyme; LTP, long‐term potentiation; NDE, neuron‐derived exosomes; NEP, neprilysin; PET, positron emission tomography; TSPO, translocator protein.

## 6 Clinical Perspectives

Although early‐phase studies of MSC‐based therapies in AD models are encouraging, translation into clinical settings is still in progress. Human trials investigating MSCs from bone marrow, adipose tissue, and umbilical cord sources have begun to report safety and feasibility, yet conclusive results regarding efficacy remain limited (X. Han et al. 2025). The path forward will require not only larger and more rigorous clinical studies but also the development of standardized protocols for exosome characterization and delivery. If these hurdles can be overcome, exosome‐based biomarkers and therapeutics may fundamentally transform both the diagnosis and treatment of AD, offering a future where earlier detection, more precise monitoring, and targeted intervention are possible (Shahlaei et al. 2025). Over the last decade, multiple clinical trials have explored the therapeutic potential of MSCs in AD, utilizing a range of stem cell sources, delivery routes, and experimental protocols. While most of these investigations remain in early stages, the accumulating evidence has provided valuable insights into feasibility and safety (Hu and Wang 2022).

In the United States, a trial involving 33 patients with AD assessed the intravenous delivery of bone marrow‐derived MSCs. Although the treatment was well tolerated, no significant cognitive improvement was reported (Brody et al. 2023). A separate American study enrolled 21 participants and tested adipose‐derived MSCs through the same administration route. This study also supported safety but was limited by its small sample size, which restricted meaningful conclusions (A Phase 1/2, Randomized, Double‐Blind, Placebo‐Controlled Study to Evaluate the Safety and Efficacy of AstroStem, Autologous Adipose Tissue Derived Mesenchymal Stem Cells, in Patients With Alzheimer's Disease 2017). South Korea has been particularly active in this field, conducting several trials with umbilical cord blood–derived MSCs (UCB‐MSCs). One study tested intracerebroventricular infusion in 45 patients and confirmed the safety of the approach, but its effect on disease progression remained uncertain (A Double‐blind, Single‐center, Phase 1/2a Clinical Trial to Evaluate the Safety and Exploratory Efficacy of Intraventricular Administrations of NEUROSTEM Versus Placebo Via an Ommaya Reservoir in Patients With Alzheimer's Disease 2014). Another Korean trial used direct intracerebral transplantation of UCB‐MSCs in nine patients; while again no major safety concerns emerged, the therapeutic benefits were weak (Open‐Label, Single‐Center, Phase 1 Clinical Trial to Evaluate the Safety and the Efficacy of NEUROTSTEM‐AD in Patients With Dementia of the Alzheimer's Type 2011).

Ongoing and planned trials are expanding these efforts. In the United States, new studies are recruiting or preparing to recruit patients to evaluate intravenous bone marrow‐derived MSCs in groups ranging from 40 to 80 participants, with an emphasis on both safety and anti‐inflammatory effects (A Phase IIa Study of Allogeneic Human Mesenchymal Stem Cells in Subjects With Mild to Moderate Dementia Due to Alzheimer's Disease 2016; A Phase 2b, Randomized, Double‐Blind, Placebo‐Controlled Study to Assess the Efficacy and Safety of AstroStem, Autologous Adipose Tissue Derived Mesenchymal Stem Cells, in Patients With Alzheimer's Disease 2020). South Korea is simultaneously running another intracerebroventricular study with UCB‐MSCs, while several smaller pilot projects in China are investigating umbilical cord‐derived MSCs delivered intravenously, though the status of these trials is not clearly reported (Exploratory Efficacy Study of NEUROSTEM in Subjects Who Control Group of NEUROSTEM Phase‐I/IIa Clinical Trial 2021; Clinical Study on the Safety and Efficacy of Umbilical Cord Mesenchymal Stem Cell Injection in the Treatment of Mild and Moderate Alzheimer's Disease 2016). Some investigations have faced setbacks; for example, a US phase I trial designed to evaluate intravenous adipose‐derived MSCs in 24 patients was terminated due to disruptions caused by the COVID‐19 pandemic (A Clinical Trial to Determine the Safety and Efficacy of Hope Biosciences Autologous Mesenchymal Stem Cell Therapy (HB‐adMSCs) for the Treatment of Alzheimer's Disease 2020).

Innovative administration routes are also being tested. A Chinese study is currently examining nasal delivery of adipose‐derived MSCs in a small group of nine patients, an approach intended to bypass invasive procedures while enhancing direct access to the central nervous system (Open‐Label, Single‐Center, Phase I/II Clinical Trial to Evaluate the Safety and the Efficacy of Exosomes Derived From Allogenic Adipose Mesenchymal Stem Cells in Patients With Mild to Moderate Dementia Due to Alzheimer's Disease 2020). In addition, a very large‐scale clinical trial has been registered in the United States, planning to enroll up to 5000 patients to examine MSC therapy safety and efficacy on a population‐wide level. Despite this progress, the field remains at an exploratory stage, and much work is needed to harmonize protocols, standardize endpoints, and establish long‐term outcome measures. Together, the trials summarized in Table 2 motivate a focused interpretation of why early studies often yield neutral cognitive outcomes and how future designs can address these limitations (Table 3) (Evaluation of the Safety, Tolerability and Efficacy of Regenerative Therapy for the Treatment of Various Chronic, and Acute Conditions 2020).

**TABLE 3:** Clinical trials of MSC‐based therapies in AD.

Country	Source of MSCs	Route of administration	Patient sample size	Key outcomes	Ref
**USA**	Bone marrow	Intravenous	33	Safe, no significant cognitive improvement	Brody et al. (2023)
**USA**	Adipose tissue	Intravenous	21	Safe, but small sample size limited conclusions	A Phase 1/2, Randomized, Double‐Blind, Placebo‐Controlled Study to Evaluate the Safety and Efficacy of AstroStem, Autologous Adipose Tissue Derived Mesenchymal Stem Cells, in Patients With Alzheimer's Disease (2017)
**South Korea**	Umbilical cord blood (UCB)	Intracerebroventricular	45	Safe; efficacy on disease progression unclear	A Double‐blind, Single‐center, Phase 1/2a Clinical Trial to Evaluate the Safety and Exploratory Efficacy of Intraventricular Administrations of NEUROSTEM Versus Placebo Via an Ommaya Reservoir in Patients With Alzheimer's Disease (2014)
**South Korea**	UCB	Intracerebral transplantation	9	Safe; weak therapeutic evidence	Open‐Label, Single‐Center, Phase 1 Clinical Trial to Evaluate the Safety and the Efficacy of NEUROTSTEM‐AD in Patients With Dementia of the Alzheimer's Type (2011)
**China**	Adipose‐derived MSCs	Intranasal	9	Innovative delivery, under evaluation	Open‐Label, Single‐Center, Phase I/II Clinical Trial to Evaluate the Safety and the Efficacy of Exosomes Derived From Allogenic Adipose Mesenchymal Stem Cells in Patients With Mild to Moderate Dementia Due to Alzheimer's Disease (2020)

### 6.1 Follow‐up and Current Status of Representative Trials

The bone marrow and adipose intravenous studies in the United States reported acceptable safety with neutral cognitive outcomes in small cohorts, and one adipose trial was terminated early due to pandemic‐related disruption. Umbilical cord blood studies in South Korea have established procedural safety with intracerebroventricular or intracerebral delivery, while effects on disease progression remain uncertain. Additional trials are recruiting to refine dose, delivery route, and target populations, including intranasal administration designed to improve brain exposure. Together, these examples illustrate feasibility, underscore the need for standardized manufacturing and endpoints, and motivate biomarker‐driven designs in prodromal stages.

### 6.2 Interpreting Neutral Clinical Outcomes

Several early trials report safety without a clear cognitive benefit. Likely contributors include small sample sizes, variability in cell source and manufacturing that alters exosome yield and cargo, suboptimal dose or frequency, systemic delivery with limited brain exposure, short follow‐up windows, heterogeneous endpoints, concomitant medications and cerebrovascular comorbidities, immunosenescence, and enrollment late in the disease continuum. These factors motivate standardized GMP manufacturing and characterization, dose finding with pharmacodynamic biomarkers, brain‐targeted delivery such as intranasal or ligand‐directed exosomes, enrichment of prodromal or mild cognitive impairment cohorts, and composite outcomes that integrate fluid biomarkers with sensitive cognitive domains.

### 6.3 Ethical and Regulatory Considerations

Translation of MSC‐ and exosome‐based approaches into clinical practice requires robust ethical oversight and operational rigor. Protocols should obtain Institutional Review Board or Research Ethics Committee approval, be prospectively registered with defined data and safety monitoring, and include clear informed consent that explains the investigational nature of exosome products, foreseeable risks, the possibility of no direct personal benefit, data and privacy safeguards including biobanking and future sample use, and the right to withdraw without penalty (Lee et al. 2024). Because many AD participants have cognitive impairment, capacity must be assessed and, where needed, consent obtained from a legally authorized representative with ongoing assent from the participant (L. Wang, Zhang, et al. 2024). Operationally, donor screening and traceability, GMP‐compliant manufacturing, standardized release testing, and chain‐of‐custody procedures are essential to limit heterogeneity and contamination (Thakur and Rai 2024). For diagnostics, blood‐based liquid biopsy strategies using brain‐derived extracellular vesicles are advancing but remain within evolving regulatory frameworks (A. Kumar, Nader, et al. 2024). In the United States, exosome‐based assays would be regulated as in vitro diagnostics or as laboratory‐developed tests under CLIA, and would require rigorous analytical and clinical validation before clinical deployment (C. Y. Ma et al. 2024). In the European Union, the In Vitro Diagnostic Regulation requires CE marking with evidence of safety, performance, and clinical utility (Humbert et al. 2025). Across Asia, regulators follow similar principles, and to date, there is no broad approval for routine clinical use of exosome‐based Alzheimer's diagnostics, reflecting a cautious stance that emphasizes validation and patient safety (Cleary et al. 2025). Convergent global expectations therefore prioritize analytical validity, clinical validity, and clinical utility, together with harmonized international guidance, standardized isolation and characterization workflows, and post‐marketing surveillance to ensure public trust and safe clinical translation (Wen et al. 2025).

## 7 Advantages and Challenges of MSC‐Based Therapies

MSCs hold a unique place in regenerative medicine because of their ability to differentiate into multiple lineages, including osteocytes, chondrocytes, and adipocytes, while also exerting strong immunomodulatory effects. These properties make them attractive for neurodegenerative disorders, where both neuronal loss and chronic inflammation drive disease progression (X. Han et al. 2025). Preclinical research has even suggested that MSCs may have antitumorigenic properties, with studies showing reduced proliferation and migration of various human cancer cell lines when cultured in MSC‐conditioned environments (Ramuta and Kreft 2022). From a practical standpoint, MSCs can be harvested from bone marrow, adipose tissue, or umbilical cord blood using relatively simple and minimally invasive techniques. Their low immunogenicity allows for transplantation across donors with a minimal risk of rejection (Karaoz et al. 2019; Al‐Ameer et al. 2025). In addition, most clinical experiences so far indicate that single MSC administrations are safe and rarely trigger adverse immune responses. Nevertheless, repeated administrations may raise concerns about alloantibody formation, which underscores the need for careful long‐term monitoring (Sanabria‐de la Torre et al. 2021).

The limitations are equally important. Despite the enthusiasm, only a small number of AD clinical trials involving MSCs have been completed, and the results from animal models do not yet provide conclusive proof of efficacy (Mastrolia et al. 2019). Furthermore, the mechanisms through which MSCs exert neuroprotective effects remain incompletely understood, with paracrine signaling, immunomodulation, and exosome release all implicated. Ethical, social, and regulatory issues also complicate the landscape (Fan et al. 2020). In the United States, the FDA has so far only approved cord blood–derived stem cell products, yet numerous clinics continue to market unregulated and expensive stem cell interventions, raising concerns about patient safety and public trust. Establishing clear international regulatory frameworks and ensuring ongoing monitoring of patients receiving stem cell–based interventions are therefore critical for advancing the field responsibly (Brinsfield et al. 2024) (Table 4).

**TABLE 4:** Advantages and challenges of MSC‐exosome therapies in AD.

Category	Advantages	Challenges	Clinical significance	Future perspective	Ref
**Biological** properties	Multipotent, immunomodulatory, anti‐inflammatory	Incomplete understanding of mechanisms	Broad therapeutic potential	Need deeper mechanistic studies	Liao et al. (2024)
**Safety** profile	Non‐tumorigenic, low immunogenicity	Repeated use may trigger alloantibodies	Safer than iPSCs/ESCs	Optimize dosing regimens	Shah et al. (2024)
**Biomarker** potential	Reflect CNS pathology, accessible in blood/saliva	Variability in exosome isolation	Early diagnosis possible	Develop standardized protocols	Shahlaei et al. (2025)
**Therapeutic** efficacy	Promote neurogenesis, reduce Aβ/tau, restore synapses	Limited clinical efficacy evidence	Disease modification possible	Combine with precision medicine	Reza‐Zaldivar et al. (2019)
**Regulatory** landscape	Minimally invasive collection	Unregulated clinics, ethical concerns	Protects patients	Establish global regulatory frameworks	Shah et al. (2024)

## 8 Conclusions

MSC‐Exos have emerged as powerful mediators of intercellular communication between MSCs and neural cells, including microglia and neurons. These nanoscale vesicles carry microRNAs, trophic factors, enzymes, and immunoregulatory molecules that collectively promote neurogenesis, reduce inflammation, and protect hippocampal neurons from damage. Notably, their immunomodulatory and neuroprotective effects are often comparable to, or even greater than, those observed with parent MSCs. Unlike whole‐cell therapies, MSC‐Exos function independently of the host microenvironment, maintaining stability in phenotype and activity across different conditions. This consistency makes them an appealing cell‐free therapeutic platform. By avoiding challenges such as cell engraftment, tumorigenicity, or immune rejection, exosome‐based therapies may overcome many of the limitations of direct stem cell transplantation. Consequently, MSC‐Exos are increasingly recognized as a potential alternative to cell‐based therapies, offering a safer, more controlled, and scalable strategy for tackling neurocognitive disorders such as AD. As clinical trials continue to refine their application, exosome‐based therapeutics may soon shift from experimental promise to clinical reality.

## Author Contributions

**Vinay Patil**: conceptualization, writing–original draft, writing–review and editing, supervision. **Bhavin Parekh**: conceptualization, writing–original draft, writing–review and editing, supervision. **Amit Sharma**: conceptualization, writing–original draft, writing–review and editing, supervision. **Husni Farah**: conceptualization, writing–original draft, writing–review and editing, supervision. **Renuka Jyothi‐S**: conceptualization, writing–original draft, writing–review and editing, supervision. **Swati Mishra**: conceptualization, writing–original draft, writing–review and editing, supervision. **Anima Nanda**: conceptualization, writing–original draft, writing–review and editing, supervision. **Shaker Al‐Hasnaawei**: conceptualization, writing–original draft, writing–review and editing, supervision. **Manoj Kumar‐Mishra**: conceptualization, writing–original draft, writing–review and editing, supervision.

## Funding

The authors have nothing to report.

## Ethics Statement

The authors have nothing to report.

## Consent

The authors have nothing to report.

## Conflicts of Interest

The authors declare no conflicts of interest.

## Data Availability

Data sharing is not applicable to this article as no datasets were generated or analyzed during the current study.

## References

[brb371112-bib-0001] Clinical Study on the Safety and Efficacy of Umbilical Cord Mesenchymal Stem Cell Injection in the Treatment of Mild and Moderate Alzheimer's Disease. 2016. https://clinicaltrials.gov/study/NCT02672306.

[brb371112-bib-0002] 2023 Alzheimer's Disease Facts and Figures . 2023. *Alzheimer's & Dementia* 19, no. 4: 1598–1695. 10.1002/alz.13016 36918389

[brb371112-bib-0003] A Clinical Trial to Determine the Safety and Efficacy of Hope Biosciences Autologous Mesenchymal Stem Cell Therapy (HB‐adMSCs) for the Treatment of Alzheimer's Disease . 2020. https://clinicaltrials.gov/study/NCT04228666.

[brb371112-bib-0004] A Double‐Blind, Single‐Center, Phase 1/2a Clinical Trial to Evaluate the Safety and Exploratory Efficacy of Intraventricular Administrations of NEUROSTEM® Versus Placebo Via an Ommaya Reservoir in Patients With Alzheimer's Disease . 2014. https://clinicaltrials.gov/study/NCT02054208.

[brb371112-bib-0005] A Phase 1/2, Randomized, Double‐Blind, Placebo‐Controlled Study to Evaluate the Safety and Efficacy of AstroStem, Autologous Adipose Tissue Derived Mesenchymal Stem Cells, in Patients With Alzheimer's Disease . 2017. https://clinicaltrials.gov/study/NCT03117738.

[brb371112-bib-0006] A Phase 2b, Randomized, Double‐Blind, Placebo‐Controlled Study to Assess the Efficacy and Safety of AstroStem, Autologous Adipose Tissue Derived Mesenchymal Stem Cells, in Patients With Alzheimer's Disease . 2020. https://clinicaltrials.gov/study/NCT04482413.

[brb371112-bib-0007] A Phase IIa Study of Allogeneic Human Mesenchymal Stem Cells in Subjects With Mild to Moderate Dementia Due to Alzheimer's Disease . 2016. https://clinicaltrials.gov/study/NCT02833792.

[brb371112-bib-0008] Abbas Raja, A. , A. Amjad , A. Choudhary , et al. 2025. “Comparative Effectiveness of Rivastigmine and Donepezil in Patients with Alzheimer's Disease: A Retrospective Cohort Study.” Cureus 17, no. 5: e83498. 40470403 10.7759/cureus.83498 PMC12135722

[brb371112-bib-0009] Al‐Ameer, H. J. , M. Zihlif , A. Maslat , et al. 2025. “Targeting the Proliferation of Glioblastoma Cells and Enhancement of Doxorubicin and Temozolomide Cytotoxicity Through Inhibition of PFKFB4 and HMOX1 Genes With siRNAs.” Scientific Reports 15, no. 1: 27861. 40739397 10.1038/s41598-025-97192-z PMC12311046

[brb371112-bib-0010] Al‐Sahlawi, F. , A. Y. M. Alabdali , S. Chinnappan , A. Al‐Samydai , and M. A. A. Maki . 2024. “Polymer‐Based Nanoparticles in Targeted Cancer Therapy: A Review.” Journal of Applied Pharmaceutical Science 14, no. 9: 057–068.

[brb371112-bib-0011] Bécot, A. , C. Volgers , and G. van Niel . 2020. “Transmissible Endosomal Intoxication: A Balance Between Exosomes and Lysosomes at the Basis of Intercellular Amyloid Propagation.” Biomedicines 8, no. 8: 272. 32759666 10.3390/biomedicines8080272 PMC7459801

[brb371112-bib-0012] Bhatti, J. S. , N. Khullar , J. Mishra , et al. 2023. “Stem Cells in the Treatment of Alzheimer's Disease—Promises and Pitfalls.” Biochimica Et Biophysica Acta (BBA)—Molecular Basis of Disease 1869, no. 6: 166712. 37030521 10.1016/j.bbadis.2023.166712

[brb371112-bib-0013] Bhome, R. , F. Del Vecchio , G. H. Lee , et al. 2018. “Exosomal MicroRNAs (exomiRs): Small Molecules With a Big Role in Cancer.” Cancer Letters 420: 228–235. 29425686 10.1016/j.canlet.2018.02.002 PMC5831981

[brb371112-bib-0014] Bloom, G. S. 2014. “Amyloid‐β and Tau: The Trigger and Bullet in Alzheimer Disease Pathogenesis.” JAMA Neurology 71, no. 4: 505–508. 24493463 10.1001/jamaneurol.2013.5847 PMC12908160

[brb371112-bib-0015] Boscher, E. , J. Hernandez‐Rapp , S. Petry , et al. 2020. “Advances and Challenges in Understanding MicroRNA Function in Tauopathies: A Case Study of miR‐132/212.” Frontiers in Neurology 11: 578720. 33117266 10.3389/fneur.2020.578720 PMC7553085

[brb371112-bib-0016] Braak, H. , D. R. Thal , E. Ghebremedhin , and K. Del Tredici . 2011. “Stages of the Pathologic Process in Alzheimer Disease: Age Categories From 1 to 100 Years.” Journal of Neuropathology & Experimental Neurology 70, no. 11: 960–969. 22002422 10.1097/NEN.0b013e318232a379

[brb371112-bib-0017] Brinsfield, T. N. , N. R. Pinson , and A. D. Levine . 2024. “The Evolution and Ongoing Challenge of Unproven Cell‐Based Interventions.” Stem Cells Translational Medicine 13, no. 9: 851–858. 39045646 10.1093/stcltm/szae050 PMC11386208

[brb371112-bib-0018] Brody, M. , M. Agronin , B. J. Herskowitz , et al. 2023. “Results and Insights From a Phase I Clinical Trial of Lomecel‐B for Alzheimer's Disease.” Alzheimer's Dementia 19, no. 1: 261–273. 10.1002/alz.12651 PMC10084163 35357079

[brb371112-bib-0019] Cai, Y. , J. Liu , B. Wang , M. Sun , and H. Yang . 2022. “Microglia in the Neuroinflammatory Pathogenesis of Alzheimer's Disease and Related Therapeutic Targets.” Frontiers in Immunology 13: 856376. 35558075 10.3389/fimmu.2022.856376 PMC9086828

[brb371112-bib-0020] Chaddha, J. , E. Blaney , A. Al‐Salahat , et al. 2025. “Trends and Disparities in Alzheimer's Disease Mortality in the United States: The Impact of COVID‐19.” NeuroSci 6, no. 1: 16. 39982268 10.3390/neurosci6010016 PMC11843863

[brb371112-bib-0021] Chavda, V. P. , A. Pandya , L. Kumar , et al. 2023. “Exosome Nanovesicles: A Potential Carrier for Therapeutic Delivery.” Nano Today 49: 101771.

[brb371112-bib-0022] Checler, F. , E. Afram , R. Pardossi‐Piquard , and I. Lauritzen . 2021. “Is γ‐Secretase a Beneficial Inactivating Enzyme of the Toxic APP C‐Terminal Fragment C99?” Journal of Biological Chemistry 296: 100489. 33662398 10.1016/j.jbc.2021.100489 PMC8027268

[brb371112-bib-0023] Chen, Y. , Y. Li , L. Huang , et al. 2021. “Antioxidative Stress: Inhibiting Reactive Oxygen Species Production as a Cause of Radioresistance and Chemoresistance.” Oxidative Medicine and Cellular Longevity 2021: 6620306. 33628367 10.1155/2021/6620306 PMC7884184

[brb371112-bib-0024] Chen, Z. R. , J. B. Huang , S. L. Yang , and F. F. Hong . 2022. “Role of Cholinergic Signaling in Alzheimer's Disease.” Molecules 27, no. 6: 1816. 35335180 10.3390/molecules27061816 PMC8949236

[brb371112-bib-0025] Cleary, J. A. , A. Kumar , S. Craft , and G. Deep . 2025. “Neuron‐Derived Extracellular Vesicles as a Liquid Biopsy for Brain Insulin Dysregulation in Alzheimer's Disease and Related Disorders.” Alzheimer's & Dementia 21, no. 2: e14497. 10.1002/alz.14497 PMC11848159 39822132

[brb371112-bib-0026] Cone, A. S. , X. Yuan , L. Sun , et al. 2021. “Mesenchymal Stem Cell‐Derived Extracellular Vesicles Ameliorate Alzheimer's Disease‐Like Phenotypes in a Preclinical Mouse Model.” Theranostics 11, no. 17: 8129–8142. 34373732 10.7150/thno.62069 PMC8344012

[brb371112-bib-0027] Coughlan, C. , K. Bruce , O. Burgy , et al. 2020. “Exosome Isolation by Ultracentrifugation and Precipitation and Techniques for Downstream Analyses.” Current Protocols in Cell Biology 88: e110. 32633898 10.1002/cpcb.110 PMC8088761

[brb371112-bib-0028] Cui, G. H. , H. D. Guo , H. Li , et al. 2019. “RVG‐Modified Exosomes Derived From Mesenchymal Stem Cells Rescue Memory Deficits by Regulating Inflammatory Responses in a Mouse Model of Alzheimer's Disease.” Immunity & Ageing 16: 10. 31114624 10.1186/s12979-019-0150-2 PMC6515654

[brb371112-bib-0029] de Godoy, M. A. , L. M. Saraiva , L. R. P. de Carvalho , et al. 2018. “Mesenchymal Stem Cells and Cell‐Derived Extracellular Vesicles Protect Hippocampal Neurons From Oxidative Stress and Synapse Damage Induced by Amyloid‐β Oligomers.” Journal of Biological Chemistry 293, no. 6: 1957–1975. 29284679 10.1074/jbc.M117.807180 PMC5808759

[brb371112-bib-0030] Dehghani, S. , O. Ocakcı , P. T. Hatipoglu , V. C. Özalp , and A. Tevlek . 2025. “Exosomes as Biomarkers and Therapeutic Agents in Neurodegenerative Diseases: Current Insights and Future Directions.” Molecular Neurobiology 62, no. 7: 9190–9215. 40095345 10.1007/s12035-025-04825-5 PMC12209394

[brb371112-bib-0031] Delshad, M. , M.‐J. Sanaei , M. H. Mohammadi , A. Sadeghi , and D. Bashash . 2025. “Exosomal Biomarkers: A Comprehensive Overview of Diagnostic and Prognostic Applications in Malignant and Non‐Malignant Disorders.” Biomolecules 15, no. 4: 587. 40305328 10.3390/biom15040587 PMC12024574

[brb371112-bib-0032] Deokate, N. , S. Acharya , R. Patil , S. M. Shaikh , and V. Karwa . 2024. “A Comprehensive Review of the Role of Stem Cells in Neuroregeneration: Potential Therapies for Neurological Disorders.” Cureus 16, no. 8: e67506. 39310492 10.7759/cureus.67506 PMC11416137

[brb371112-bib-0033] De Sousa, K. P. , I. Rossi , M. Abdullahi , M. I. Ramirez , D. Stratton , and J. M. Inal . 2023. “Isolation and Characterization of Extracellular Vesicles and Future Directions in Diagnosis and Therapy.” Wiley Interdisciplinary Reviews: Nanomedicine and Nanobiotechnology 15, no. 1: e1835. 35898167 10.1002/wnan.1835 PMC10078256

[brb371112-bib-0034] DeTure, M. A. , and D. W. Dickson . 2019. “The Neuropathological Diagnosis of Alzheimer's Disease.” Molecular Neurodegeneration 14, no. 1: 32. 31375134 10.1186/s13024-019-0333-5 PMC6679484

[brb371112-bib-0035] Doyle, L. M. , and M. Z. Wang . 2019. “Overview of Extracellular Vesicles, Their Origin, Composition, Purpose, and Methods for Exosome Isolation and Analysis.” Cells 8, no. 7: 727. 31311206 10.3390/cells8070727 PMC6678302

[brb371112-bib-0036] Edwards, G. A., III , N. Gamez , G. Escobedo, Jr. , O. Calderon , and I. Moreno‐Gonzalez . 2019. “Modifiable Risk Factors for Alzheimer's Disease.” Frontiers in Aging Neuroscience 11: 146. 31293412 10.3389/fnagi.2019.00146 PMC6601685

[brb371112-bib-0037] Evaluation of the Safety, Tolerability and Efficacy of Regenerative Therapy for the Treatment of Various Chronic and Acute Conditions . 2020. https://clinicaltrials.gov/study/NCT04684602.

[brb371112-bib-0038] Exploratory Efficacy Study of NEUROSTEM® in Subjects Who Control Group of NEUROSTEM® Phase‐I/IIa Clinical Trial . 2021. https://clinicaltrials.gov/study/NCT04954534.

[brb371112-bib-0039] Fan, X. L. , Y. Zhang , X. Li , and Q. L. Fu . 2020. “Mechanisms Underlying the Protective Effects of Mesenchymal Stem Cell‐Based Therapy.” Cellular and Molecular Life Sciences 77, no. 14: 2771–2794. 31965214 10.1007/s00018-020-03454-6 PMC7223321

[brb371112-bib-0040] Fayazi, N. , M. Sheykhhasan , S. Soleimani Asl , and R. Najafi . 2021. “Stem Cell‐Derived Exosomes: A New Strategy of Neurodegenerative Disease Treatment.” Molecular Neurobiology 58, no. 7: 3494–3514. 33745116 10.1007/s12035-021-02324-x PMC7981389

[brb371112-bib-0041] Ghadami, S. , and K. Dellinger . 2023. “The Lipid Composition of Extracellular Vesicles: Applications in Diagnostics and Therapeutic Delivery.” Frontiers in Molecular Biosciences 10: 1198044. 37520326 10.3389/fmolb.2023.1198044 PMC10381967

[brb371112-bib-0042] Goetzl, E. J. , J. B. Schwartz , E. L. Abner , G. A. Jicha , and D. Kapogiannis . 2018. “High Complement Levels in Astrocyte‐Derived Exosomes of Alzheimer Disease.” Annals of Neurology 83, no. 3: 544–552. 29406582 10.1002/ana.25172 PMC5867263

[brb371112-bib-0043] Govindpani, K. , L. G. McNamara , N. R. Smith , et al. 2019. “Vascular Dysfunction in Alzheimer's Disease: A Prelude to the Pathological Process or a Consequence of It?” Journal of Clinical Medicine 8, no. 5: 651. 31083442 10.3390/jcm8050651 PMC6571853

[brb371112-bib-0044] Greenberg, S. M. , B. J. Bacskai , M. Hernandez‐Guillamon , J. Pruzin , R. Sperling , and S. J. van Veluw . 2020. “Cerebral Amyloid Angiopathy and Alzheimer Disease—One Peptide, Two Pathways.” Nature Reviews Neurology 16, no. 1: 30–42. 31827267 10.1038/s41582-019-0281-2 PMC7268202

[brb371112-bib-0045] Grewal, A. , S. Raikundalia , J. Zaia , and M. K. Sethi . 2025. “Overview of Proteomic Analysis of Amyloid Plaques and Neurofibrillary Tangles in Alzheimer's Disease.” Biomolecules 15, no. 9: 1310. 41008617 10.3390/biom15091310 PMC12467479

[brb371112-bib-0046] Grimm, M. O. , J. Mett , C. P. Stahlmann , V. J. Haupenthal , V. C. Zimmer , and T. Hartmann . 2013. “Neprilysin and Aβ Clearance: Impact of the APP Intracellular Domain in NEP Regulation and Implications in Alzheimer's Disease.” Frontiers in Aging Neuroscience 5: 98. 24391587 10.3389/fnagi.2013.00098 PMC3870290

[brb371112-bib-0047] Guha, D. , D. R. Lorenz , V. Misra , S. Chettimada , S. Morgello , and D. Gabuzda . 2019. “Proteomic Analysis of Cerebrospinal Fluid Extracellular Vesicles Reveals Synaptic Injury, Inflammation, and Stress Response Markers in HIV Patients With Cognitive Impairment.” Journal of Neuroinflammation 16, no. 1: 254. 31805958 10.1186/s12974-019-1617-y PMC6896665

[brb371112-bib-0048] Gurung, S. , D. Perocheau , L. Touramanidou , and J. Baruteau . 2021. “The Exosome Journey: From Biogenesis to Uptake and Intracellular Signalling.” Cell Communication and Signaling 19, no. 1: 47. 33892745 10.1186/s12964-021-00730-1 PMC8063428

[brb371112-bib-0049] Hampel, H. , Y. Hu , J. Cummings , et al. 2023. “Blood‐Based Biomarkers for Alzheimer's Disease: Current State and Future Use in a Transformed Global Healthcare Landscape.” Neuron 111, no. 18: 2781–2799. 37295421 10.1016/j.neuron.2023.05.017 PMC10720399

[brb371112-bib-0050] Han, Q. F. , W. J. Li , K. S. Hu , et al. 2022. “Exosome Biogenesis: Machinery, Regulation, and Therapeutic Implications in Cancer.” Molecular Cancer 21, no. 1: 207. 36320056 10.1186/s12943-022-01671-0 PMC9623991

[brb371112-bib-0051] Han, X. , R. Liao , X. Li , et al. 2025. “Mesenchymal Stem Cells in Treating Human Diseases: Molecular Mechanisms and Clinical Studies.” Signal Transduction and Targeted Therapy 10, no. 1: 262. 40841367 10.1038/s41392-025-02313-9 PMC12371117

[brb371112-bib-0052] Horbay, R. , A. Hamraghani , L. Ermini , S. Holcik , S. T. Beug , and B. Yeganeh . 2022. “Role of Ceramides and Lysosomes in Extracellular Vesicle Biogenesis, Cargo Sorting and Release.” International Journal of Molecular Sciences 23, no. 23: 15317. 36499644 10.3390/ijms232315317 PMC9735581

[brb371112-bib-0053] Hu, J. , and X. Wang . 2022. “Alzheimer's Disease: From Pathogenesis to Mesenchymal Stem Cell Therapy—Bridging the Missing Link.” Frontiers in Cellular Neuroscience 15: 811852. 35197824 10.3389/fncel.2021.811852 PMC8859419

[brb371112-bib-0054] Huang, L. , M. Liu , Z. Li , B. Li , J. Wang , and K. Zhang . 2025. “Systematic Review of Amyloid‐Beta Clearance Proteins From the Brain to the Periphery: Implications for Alzheimer's Disease Diagnosis and Therapeutic Targets.” Neural Regeneration Research 20, no. 12: 3574–3590. 39820231 10.4103/NRR.NRR-D-24-00865 PMC11974662

[brb371112-bib-0055] Humbert, C. , C. Cordier , I. Drut , et al. 2025. “GMP‐Compliant Process for the Manufacturing of an Extracellular Vesicles‐Enriched Secretome Product Derived from Cardiovascular Progenitor Cells Suitable for a Phase I Clinical Trial.” Journal of Extracellular Vesicles 14, no. 8: e70145. 40831309 10.1002/jev2.70145 PMC12365392

[brb371112-bib-0056] Huo, L. , X. Du , X. Li , S. Liu , and Y. Xu . 2021. “The Emerging Role of Neural Cell‐Derived Exosomes in Intercellular Communication in Health and Neurodegenerative Diseases.” Frontiers in Neuroscience 15: 738442. 34531720 10.3389/fnins.2021.738442 PMC8438217

[brb371112-bib-0057] Hussen, B. M. , M. Taheri , R. K. Yashooa , et al. 2024. “Revolutionizing Medicine: Recent Developments and Future Prospects in Stem‐Cell Therapy.” International Journal of Surgery 110, no. 12: 8002–8024. 39497543 10.1097/JS9.0000000000002109 PMC11634165

[brb371112-bib-0058] Kamatham, P. T. , R. Shukla , D. K. Khatri , and L. K. Vora . 2024. “Pathogenesis, Diagnostics, and Therapeutics for Alzheimer's Disease: Breaking the Memory Barrier.” Ageing Research Reviews 101: 102481. 39236855 10.1016/j.arr.2024.102481

[brb371112-bib-0059] Karaoz, E. , E. Sun , and C. S. Demir . 2019. “Mesenchymal Stem Cell‐Derived Exosomes Do Not Promote the Proliferation of Cancer Cells in Vitro.” International Journal of Physiology, Pathophysiology and Pharmacology 11: 177–189. 31523364 PMC6737426

[brb371112-bib-0060] Kulus, M. , R. Sibiak , K. Stefańska , et al. 2021. “Mesenchymal Stem/Stromal Cells Derived From Human and Animal Perinatal Tissues—Origins, Characteristics, Signaling Pathways, and Clinical Trials.” Cells 10, no. 12: 3278. 34943786 10.3390/cells10123278 PMC8699543

[brb371112-bib-0061] Kumar, A. , M. A. Nader , and G. Deep . 2024. “Emergence of Extracellular Vesicles as “Liquid Biopsy” for Neurological Disorders: Boom or Bust.” Pharmacological Reviews 76, no. 2: 199–227. 38351075 10.1124/pharmrev.122.000788 PMC10877757

[brb371112-bib-0062] Kumar, A. , J. Sidhu , F. Lui , and J. W. Tsao . 2024. “Alzheimer disease.” In StatPearls. StatPearls Publishing. 29763097

[brb371112-bib-0063] Kumar, M. A. , S. K. Baba , H. Q. Sadida , et al. 2024. “Extracellular Vesicles as Tools and Targets in Therapy for Diseases.” Signal Transduction and Targeted Therapy 9, no. 1: 27. 38311623 10.1038/s41392-024-01735-1 PMC10838959

[brb371112-bib-0064] Lee, K. W. A. , L. K. W. Chan , L. C. Hung , L. K. W. Phoebe , Y. Park , and K. H. Yi . 2024. “Clinical Applications of Exosomes: A Critical Review.” International Journal of Molecular Sciences 25, no. 14: 7794. 39063033 10.3390/ijms25147794 PMC11277529

[brb371112-bib-0065] Lei, X. , L. Lei , Z. Zhang , Z. Zhang , and Y. Cheng . 2015. “Downregulated miR‐29c Correlates With Increased BACE1 Expression in Sporadic Alzheimer's Disease.” International Journal of Clinical and Experimental Pathology 8, no. 2: 1565–1574. 25973041 PMC4396232

[brb371112-bib-0066] Li, M. , H. Chen , and M. Zhu . 2022. “Mesenchymal Stem Cells for Regenerative Medicine in Central Nervous System.” Frontiers in Neuroscience 16: 1068114. 36583105 10.3389/fnins.2022.1068114 PMC9793714

[brb371112-bib-0067] Li, X. , A. L. Corbett , E. Taatizadeh , et al. 2019. “Challenges and Opportunities in Exosome Research—Perspectives From Biology, Engineering, and Cancer Therapy.” APL Bioengineering 3, no. 1: 011503. 31069333 10.1063/1.5087122 PMC6481742

[brb371112-bib-0068] Liao, H.‐J. , Y.‐P. Yang , Y.‐H. Liu , et al. 2024. “Harnessing the Potential of Mesenchymal Stem Cells–Derived Exosomes in Degenerative Diseases.” Regenerative Therapy 26: 599–610. 39253597 10.1016/j.reth.2024.08.001 PMC11382214

[brb371112-bib-0069] Liu, S. , M. Fan , J.‐X. Xu , et al. 2022. “Exosomes Derived From Bone‐Marrow Mesenchymal Stem Cells Alleviate Cognitive Decline in AD‐Like Mice by Improving BDNF‐Related Neuropathology.” Journal of Neuroinflammation 19, no. 1: 35. 35130907 10.1186/s12974-022-02393-2 PMC8822863

[brb371112-bib-0070] Ma, C. Y. , Y. Zhai , C. T. Li , et al. 2024. “Translating Mesenchymal Stem Cell and Their Exosome Research Into GMP Compliant Advanced Therapy Products: Promises, Problems and Prospects.” Medicinal Research Reviews 44, no. 3: 919–938. 38095832 10.1002/med.22002

[brb371112-bib-0071] Ma, Z. J. , J. J. Yang , Y. B. Lu , Z. Y. Liu , and X. X. Wang . 2020. “Mesenchymal Stem Cell‐Derived Exosomes: Toward Cell‐Free Therapeutic Strategies in Regenerative Medicine.” World Journal of Stem Cells 12, no. 8: 814–840. 32952861 10.4252/wjsc.v12.i8.814 PMC7477653

[brb371112-bib-0072] Manuel, G. E. , T. Johnson , and D. Liu . 2017. “Therapeutic Angiogenesis of Exosomes for Ischemic Stroke.” International Journal of Physiology, Pathophysiology and Pharmacology 9, no. 6: 188–191. 29348795 PMC5770515

[brb371112-bib-0073] Mastrolia, I. , E. M. Foppiani , A. Murgia , et al. 2019. “Challenges in Clinical Development of Mesenchymal Stromal/Stem Cells: Concise Review.” Stem Cells Translational Medicine 8, no. 11: 1135–1148. 31313507 10.1002/sctm.19-0044 PMC6811694

[brb371112-bib-0074] Matoba, S. , and Y. Zhang . 2018. “Somatic Cell Nuclear Transfer Reprogramming: Mechanisms and Applications.” Cell Stem Cell 23, no. 4: 471–485. 30033121 10.1016/j.stem.2018.06.018 PMC6173619

[brb371112-bib-0075] Mavroudis, I. , I. M. Balmus , A. Ciobica , M. N. Nicoara , A. C. Luca , and D. O. Palade . 2023. “The Role of Microglial Exosomes and miR‐124‐3p in Neuroinflammation and Neuronal Repair After Traumatic Brain Injury.” Life 13, no. 9: 1924. 37763327 10.3390/life13091924 PMC10532687

[brb371112-bib-0076] Mehrnoosh, F. , D. Rezaei , S. A. Pakmehr , et al. 2025. “The Role of *Panax ginseng* in Neurodegenerative Disorders: Mechanisms, Benefits, and Future Directions.” Metabolic Brain Disease 40, no. 4: 183. 40232582 10.1007/s11011-025-01610-0

[brb371112-bib-0077] Miron, R. J. , N. E. Estrin , A. Sculean , and Y. Zhang . 2024. “Understanding Exosomes: Part 2—Emerging Leaders in Regenerative Medicine.” Periodontology 2000 94, no. 1: s257–414. 38591622 10.1111/prd.12561

[brb371112-bib-0078] Modat, M. , D. M. Cash , L. Dos Santos Canas , M. Bocchetta , and S. Ourselin . 2023. “Machine Learning for Alzheimer's Disease and Related Dementias.” In Machine Learning for Brain Disorders, 807–846. Springer. 37988534

[brb371112-bib-0079] Mukerjee, N. , A. Bhattacharya , S. Maitra , et al. 2025. “Exosome Isolation and Characterization for Advanced Diagnostic and Therapeutic Applications.” Materials Today Bio 31: 101613. 10.1016/j.mtbio.2025.101613 PMC11950786 40161926

[brb371112-bib-0080] Nasirishargh, A. , P. Kumar , L. Ramasubramanian , et al. 2021. “Exosomal microRNAs From Mesenchymal Stem/Stromal Cells: Biology and Applications in Neuroprotection.” World Journal of Stem Cells 13, no. 7: 776–794. 34367477 10.4252/wjsc.v13.i7.776 PMC8316862

[brb371112-bib-0081] Numakawa, T. , and R. Kajihara . 2023. “Involvement of Brain‐Derived Neurotrophic Factor Signaling in the Pathogenesis of Stress‐Related Brain Diseases.” Frontiers in Molecular Neuroscience 16: 1247422. 37781095 10.3389/fnmol.2023.1247422 PMC10537938

[brb371112-bib-0082] Oh, S. H. , H. N. Kim , H.‐J. Park , J. Y. Shin , and P. H. Lee . 2015. “Mesenchymal Stem Cells Increase Hippocampal Neurogenesis and Neuronal Differentiation by Enhancing the Wnt Signaling Pathway in an Alzheimer's Disease Model.” Cell Transplantation 24, no. 6: 1097–1109. 24612635 10.3727/096368914X679237

[brb371112-bib-0083] Open‐Label, Single‐Center, Phase 1 Clinical Trial to Evaluate the Safety and the Efficacy of NEUROTSTEM®‐AD in Patients With Dementia of the Alzheimer's Type . 2011. https://clinicaltrials.gov/study/NCT01297218.

[brb371112-bib-0084] Open‐Label, Single‐Center, Phase I/II Clinical Trial to Evaluate the Safety and the Efficacy of Exosomes Derived From Allogenic Adipose Mesenchymal Stem Cells in Patients With Mild to Moderate Dementia Due to Alzheimer's Disease . 2020. https://clinicaltrials.gov/study/NCT04388982.

[brb371112-bib-0085] Oveili, E. , S. Vafaei , H. Bazavar , et al. 2023. “The Potential Use of Mesenchymal Stem Cells‐Derived Exosomes as microRNAs Delivery Systems in Different Diseases.” Cell Communication and Signaling 21, no. 1: 20. 36690996 10.1186/s12964-022-01017-9 PMC9869323

[brb371112-bib-0086] Passeri, E. , K. Elkhoury , M. Morsink , et al. 2022. “Alzheimer's Disease: Treatment Strategies and Their Limitations.” International Journal of Molecular Sciences 23, no. 22: 13954. 36430432 10.3390/ijms232213954 PMC9697769

[brb371112-bib-0087] Pinky, S. Gupta, V. Krishnakumar, Y. Sharma, A. K. Dinda, and S. Mohanty . 2021. “Mesenchymal Stem Cell Derived Exosomes: A Nano Platform for Therapeutics and Drug Delivery in Combating COVID‐19.” Stem Cell Reviews and Reports 17, no. 1: 33–43. 32661867 10.1007/s12015-020-10002-z PMC7357441

[brb371112-bib-0088] Planat‐Benard, V. , A. Varin , and L. Casteilla . 2021. “MSCs and Inflammatory Cells Crosstalk in Regenerative Medicine: Concerted Actions for Optimized Resolution Driven by Energy Metabolism.” Frontiers in Immunology 12: 626755. 33995350 10.3389/fimmu.2021.626755 PMC8120150

[brb371112-bib-0089] Qin, C. , L. Bai , Y. Li , and K. Wang . 2022. “The Functional Mechanism of Bone Marrow‐Derived Mesenchymal Stem Cells in the Treatment of Animal Models With Alzheimer's Disease: Crosstalk Between Autophagy and Apoptosis.” Stem Cell Research & Therapy 13, no. 1: 90. 35241159 10.1186/s13287-022-02765-8 PMC8895531

[brb371112-bib-0090] Qiu, C. , M. Kivipelto , and E. Von Strauss . 2009. “Epidemiology of Alzheimer's Disease: Occurrence, Determinants, and Strategies Toward Intervention.” Dialogues in Clinical Neuroscience 11, no. 2: 111–128. 19585947 10.31887/DCNS.2009.11.2/cqiu PMC3181909

[brb371112-bib-0091] Ramuta, T. , and M. E. Kreft . 2022. “Mesenchymal Stem/Stromal Cells May Decrease Success of Cancer Treatment by Inducing Resistance to Chemotherapy in Cancer Cells.” Cancers 14, no. 15: 3761. 35954425 10.3390/cancers14153761 PMC9367361

[brb371112-bib-0092] Rawat, P. , U. Sehar , J. Bisht , A. Selman , J. Culberson , and P. H. Reddy . 2022. “Phosphorylated Tau in Alzheimer's Disease and Other Tauopathies.” International Journal of Molecular Sciences 23, no. 21: 12841. 36361631 10.3390/ijms232112841 PMC9654278

[brb371112-bib-0093] Regmi, S. , D. D. Liu , M. Shen , et al. 2022. “Mesenchymal Stromal Cells for the Treatment of Alzheimer's Disease: Strategies and Limitations.” Frontiers in Molecular Neuroscience 15: 1011225. 36277497 10.3389/fnmol.2022.1011225 PMC9584646

[brb371112-bib-0094] Reza‐Zaldivar, E. E. , M. A. Hernández‐Sapiéns , Y. K. Gutiérrez‐Mercado , et al. 2019. “Mesenchymal Stem Cell‐Derived Exosomes Promote Neurogenesis and Cognitive Function Recovery in a Mouse Model of Alzheimer's Disease.” Neural Regeneration Research 14, no. 9: 1626–1634. 31089063 10.4103/1673-5374.255978 PMC6557105

[brb371112-bib-0095] Romito, A. , and G. Cobellis . 2016. “Pluripotent Stem Cells: Current Understanding and Future Directions.” Stem Cells International 2016: 9451492. 26798367 10.1155/2016/9451492 PMC4699068

[brb371112-bib-0096] Safiri, S. , A. Ghaffari Jolfayi , A. Fazlollahi , et al. 2024. “Alzheimer's Disease: A Comprehensive Review of Epidemiology, Risk Factors, Symptoms Diagnosis, Management, Caregiving, Advanced Treatments and Associated Challenges.” Frontiers in Medicine 11: 1474043. 39736972 10.3389/fmed.2024.1474043 PMC11682909

[brb371112-bib-0097] Sanabria‐de la Torre, R. , M. I. Quiñones‐Vico , A. Fernández‐González , et al. 2021. “Alloreactive Immune Response Associated to Human Mesenchymal Stromal Cells Treatment: A Systematic Review.” Journal of Clinical Medicine 10, no. 13: 2991. 34279481 10.3390/jcm10132991 PMC8269175

[brb371112-bib-0098] Shah, S. , H. M. Mansour , T. M. Aguilar , and B. Lucke‐Wold . 2024. “Mesenchymal Stem Cell‐Derived Exosomes as a Neuroregeneration Treatment for Alzheimer's Disease.” Biomedicines 12, no. 9: 2113. 39335626 10.3390/biomedicines12092113 PMC11428860

[brb371112-bib-0099] Shahlaei, M. , H. Afkhami , A. Ahmadieh‐Yazdi , et al. 2025. “Exosomes in Alzheimer's Disease: from Pathogenesis to Therapeutics—A Comprehensive Review of Diagnostic and Drug Delivery Applications.” Biomedicine & Pharmacotherapy 192: 118548. 40987204 10.1016/j.biopha.2025.118548

[brb371112-bib-0100] Shao, M. , M. Rahmdel , S. K. Shayan , et al. 2025. “Advancements in Biomaterials for Stem Cell Differentiation.” Stem Cell Reviews and Reports 21, no. 5: 1299–1310. 40257542 10.1007/s12015-025-10879-8

[brb371112-bib-0101] Sheppard, O. , and M. Coleman . 2020. Alzheimer's Disease: Etiology, Neuropathology and Pathogenesis. Exon Publications. 33400468

[brb371112-bib-0102] Shiau, J. P. , Y. T. Chuang , Y. B. Cheng , et al. 2022. “Impacts of Oxidative Stress and PI3K/AKT/mTOR on Metabolism and the Future Direction of Investigating Fucoidan‐Modulated Metabolism.” Antioxidants 11, no. 9: 1845. 35624775 10.3390/antiox11050911 PMC9137824

[brb371112-bib-0103] Slota, J. A. , and S. A. Booth . 2019. “MicroRNAs in Neuroinflammation: Implications in Disease Pathogenesis, Biomarker Discovery and Therapeutic Applications.” Non‐coding RNA 5, no. 2: 35. 31022830 10.3390/ncrna5020035 PMC6632112

[brb371112-bib-0104] Soliman, H. M. , G. A. Ghonaim , S. M. Gharib , et al. 2021. “Exosomes in Alzheimer's Disease: From Being Pathological Players to Potential Diagnostics and Therapeutics.” International Journal of Molecular Sciences 22, no. 19: 10794. 34639135 10.3390/ijms221910794 PMC8509246

[brb371112-bib-0105] Staffaroni, A. M. , F. M. Elahi , D. McDermott , et al. 2017. “Neuroimaging in Dementia.” Seminars in Neurology 37, no. 5: 510–537. 29207412 10.1055/s-0037-1608808 PMC5823524

[brb371112-bib-0106] Sun, Y. , G. Liu , K. Zhang , Q. Cao , T. Liu , and J. Li . 2021. “Mesenchymal Stem Cells‐Derived Exosomes for Drug Delivery.” Stem Cell Research & Therapy 12, no. 1: 561. 34717769 10.1186/s13287-021-02629-7 PMC8557580

[brb371112-bib-0107] Tan, F. , X. Li , Z. Wang , J. Li , K. Shahzad , and J. Zheng . 2024. “Clinical Applications of Stem Cell‐Derived Exosomes.” Signal Transduction and Targeted Therapy 9, no. 1: 17. 38212307 10.1038/s41392-023-01704-0 PMC10784577

[brb371112-bib-0108] Tenchov, R. , J. M. Sasso , and Q. A. Zhou . 2024. “Alzheimer's Disease: Exploring the Landscape of Cognitive Decline.” ACS Chemical Neuroscience 15, no. 21: 3800–3827. 39392435 10.1021/acschemneuro.4c00339 PMC11587518

[brb371112-bib-0109] Thakur, A. , and D. Rai . 2024. “Global Requirements for Manufacturing and Validation of Clinical Grade Extracellular Vesicles.” Journal of Liquid Biopsy 6: 100278. 40027307 10.1016/j.jlb.2024.100278 PMC11863704

[brb371112-bib-0110] Volarevic, V. , B. S. Markovic , M. Gazdic , et al. 2018. “Ethical and Safety Issues of Stem Cell‐Based Therapy.” International Journal of Medical Sciences 15, no. 1: 36–45. 29333086 10.7150/ijms.21666 PMC5765738

[brb371112-bib-0111] Wa, Q. , Y. Luo , Y. Tang , et al. 2024. “Mesoporous Bioactive Glass‐Enhanced MSC‐Derived Exosomes Promote Bone Regeneration and Immunomodulation In Vitro and In Vivo.” Journal of Orthopaedic Translation 49: 264–282. 39524151 10.1016/j.jot.2024.09.009 PMC11550139

[brb371112-bib-0112] Wan, T. , Y. Huang , X. Gao , W. Wu , and W. Guo . 2022. “Microglia Polarization: A Novel Target of Exosome for Stroke Treatment.” Frontiers in Cell and Developmental Biology 10: 842320. 35356292 10.3389/fcell.2022.842320 PMC8959940

[brb371112-bib-0113] Wang, C. , Y. Chen , S. Hu , and X. Liu . 2023. “Insights Into the Function of ESCRT and Its Role in Enveloped Virus Infection.” Frontiers in Microbiology 14: 1261651. 37869652 10.3389/fmicb.2023.1261651 PMC10587442

[brb371112-bib-0114] Wang, L. , X. Zhang , Z. Yang , et al. 2024. “Extracellular Vesicles: Biological Mechanisms and Emerging Therapeutic Opportunities in Neurodegenerative Diseases.” Translational Neurodegeneration 13, no. 1: 60. 39643909 10.1186/s40035-024-00453-6 PMC11622582

[brb371112-bib-0115] Wang, W. X. , B. W. Rajeev , A. J. Stromberg , et al. 2008. “The Expression of microRNA miR‐107 Decreases Early in Alzheimer's Disease and May Accelerate Disease Progression Through Regulation of Beta‐Site Amyloid Precursor Protein‐Cleaving Enzyme 1.” Journal of Neuroscience 28, no. 5: 1213–1223. 18234899 10.1523/JNEUROSCI.5065-07.2008 PMC2837363

[brb371112-bib-0116] Wang, X. , Y. Xie , G. Chen , Y. Lu , D. Wang , and L. Zhu . 2023. “Intermittent Hypoxia Therapy Ameliorates Beta‐Amyloid Pathology via TFEB‐Mediated Autophagy in Murine Alzheimer's Disease.” Journal of Neuroinflammation 20, no. 1: 240. 37864249 10.1186/s12974-023-02931-6 PMC10588168

[brb371112-bib-0117] Wang, Y. , T. Xiao , C. Zhao , and G. Li . 2024. “The Regulation of Exosome Generation and Function in Physiological and Pathological Processes.” International Journal of Molecular Sciences 25, no. 1: 255. 10.3390/ijms25010255 PMC10779122 38203424

[brb371112-bib-0118] Wang, Y.‐T. , and H. Yuan . 2022. “Research Progress of Endogenous Neural Stem Cells in Spinal Cord Injury.” Ibrain 8, no. 2: 199–209. 37786888 10.1002/ibra.12048 PMC10529172

[brb371112-bib-0119] Wei, W. , Z. Y. Wang , L. N. Ma , T. T. Zhang , Y. Cao , and H. Li . 2020. “MicroRNAs in Alzheimer's Disease: Function and Potential Applications as Diagnostic Biomarkers.” Frontiers in Molecular Neuroscience 13: 160. 32973449 10.3389/fnmol.2020.00160 PMC7471745

[brb371112-bib-0120] Wen, C. , H. Ma , L. Xu , et al. 2025. “Recent Advances in the Clinical Application of Exosomes for Disease Diagnosis and Therapeutic Strategies.” International Journal of Surgery 111, no. 7: 4609–4628. 40387699 10.1097/JS9.0000000000002518

[brb371112-bib-0121] Winston, C. N. , E. J. Goetzl , J. C. Akers , et al. 2016. “Prediction of Conversion From Mild Cognitive Impairment to Dementia With Neuronally Derived Blood Exosome Protein Profile.” Alzheimer's & Dementia 3: 63–72. 10.1016/j.dadm.2016.04.001 PMC4925777 27408937

[brb371112-bib-0122] Wuputra, K. , C. C. Ku , D. C. Wu , Y. C. Lin , S. Saito , and K. K. Yokoyama . 2020. “Prevention of Tumor Risk Associated With the Reprogramming of Human Pluripotent Stem Cells.” Journal of Experimental & Clinical Cancer Research 39, no. 1: 100. 32493501 10.1186/s13046-020-01584-0 PMC7268627

[brb371112-bib-0123] Xia, X. , Y. Wang , Y. Huang , H. Zhang , H. Lu , and J. C. Zheng . 2019. “Exosomal miRNAs in Central Nervous System Diseases: Biomarkers, Pathological Mediators, Protective Factors and Therapeutic Agents.” Progress in Neurobiology 183: 101694. 31542363 10.1016/j.pneurobio.2019.101694 PMC7323939

[brb371112-bib-0124] Xiao, Q. , P. Yan , X. Ma , et al. 2015. “Neuronal‐Targeted TFEB Accelerates Lysosomal Degradation of APP, Reducing Aβ Generation and Amyloid Plaque Pathogenesis.” Journal of Neuroscience 35, no. 35: 12137–12151. 26338325 10.1523/JNEUROSCI.0705-15.2015 PMC4556784

[brb371112-bib-0125] Yadav, A. , A. Nandy , A. Sharma , and S. Ghatak . 2024. “Exosome Mediated Cell‐Cell Crosstalk in Tissue Injury and Repair.” In Intercellular and Interorganellar Transfer and Communication in Biology and Medicine, 249–297. Springer. 10.1007/978-3-031-62036-2_12 PMC12099227 39242383

[brb371112-bib-0126] Yakubovich, E. , A. Polischouk , and V. Evtushenko . 2022. “Principles and Problems of Exosome Isolation From Biological Fluids.” Biochemistry (Moscow), Supplement Series A: Membrane and Cell Biology 16: 115–126. 35730027 10.1134/S1990747822030096 PMC9202659

[brb371112-bib-0127] Yari, H. , M. V. Mikhailova , M. Mardasi , et al. 2022. “Emerging Role of Mesenchymal Stromal Cells (MSCs)‐Derived Exosome in Neurodegeneration‐Associated Conditions: A Groundbreaking Cell‐Free Approach.” Stem Cell Research & Therapy 13, no. 1: 423. 35986375 10.1186/s13287-022-03122-5 PMC9389725

[brb371112-bib-0128] Youssef, E. , D. Palmer , B. Fletcher , and R. Vaughn . 2025. “Exosomes in Precision Oncology and Beyond: From Bench to Bedside in Diagnostics and Therapeutics.” Cancers 17, no. 6: 940. 40149276 10.3390/cancers17060940 PMC11940788

[brb371112-bib-0129] Yu, H. , J. Zhang , L. Yang , et al. 2025. “MSC‐Derived Exosomes Injectable Hyaluronic Acid Hydrogel for Enhanced Chronic Wound Healing.” Journal of Controlled Release 385: 113985. 40581219 10.1016/j.jconrel.2025.113985

[brb371112-bib-0130] Yu, Y. , Z. Wang , Z. Chai , S. Ma , A. Li , and Y. Li . 2025. “Central Nervous System‐Derived Extracellular Vesicles as Biomarkers in Alzheimer's Disease.” International Journal of Molecular Sciences 26, no. 17: 8272. 40943193 10.3390/ijms26178272 PMC12428116

[brb371112-bib-0131] Zhang, M. , and Z. Bian . 2021. “Alzheimer's Disease and microRNA‐132: A Widespread Pathological Factor and Potential Therapeutic Target.” Frontiers in Neuroscience 15: 687973. 34108863 10.3389/fnins.2021.687973 PMC8180577

[brb371112-bib-0132] Zhang, W. , D. Xiao , Q. Mao , and H. Xia . 2023. “Role of Neuroinflammation in Neurodegeneration Development.” Signal Transduction and Targeted Therapy 8, no. 1: 267. 37433768 10.1038/s41392-023-01486-5 PMC10336149

[brb371112-bib-0133] Zhang, X. , Z. Fu , L. Meng , M. He , and Z. Zhang . 2018. “The Early Events That Initiate β‐Amyloid Aggregation in Alzheimer's Disease.” Frontiers in Aging Neuroscience 10: 359. 30542277 10.3389/fnagi.2018.00359 PMC6277872

[brb371112-bib-0134] Zhang, X. , J. E. Medow , B. J. Iskandar , et al. 2017. “Invasive and Noninvasive Means of Measuring Intracranial Pressure: A Review.” Physiological Measurement 38, no. 8: R143. 28489610 10.1088/1361-6579/aa7256

[brb371112-bib-0135] Zhao, Y. , Y. Gu , Q. Zhang , H. Liu , and Y. Liu . 2023. “The Potential Roles of Exosomes Carrying APP and Tau Cleavage Products in Alzheimer's Disease.” Journal of Clinical Medicine 12, no. 5: 1883. 36902671 10.3390/jcm12051883 PMC10003549

